# Estimation of Subglottal Pressure, Vocal Fold Collision Pressure, and Intrinsic Laryngeal Muscle Activation From Neck-Surface Vibration Using a Neural Network Framework and a Voice Production Model

**DOI:** 10.3389/fphys.2021.732244

**Published:** 2021-09-01

**Authors:** Emiro J. Ibarra, Jesús A. Parra, Gabriel A. Alzamendi, Juan P. Cortés, Víctor M. Espinoza, Daryush D. Mehta, Robert E. Hillman, Matías Zañartu

**Affiliations:** ^1^Department of Electronic Engineering, Universidad Técnica Federico Santa María, Valparaíso, Chile; ^2^School of Electrical Engineering, University of the Andes, Mérida, Venezuela; ^3^Institute for Research and Development on Bioengineering and Bioinformatics, Consejo Nacional de Investigaciones Científicas y Técnicas - Universidad Nacional de Entre Ríos, Oro Verde, Argentina; ^4^Center for Laryngeal Surgery and Voice Rehabilitation Laboratory, Massachusetts General Hospital–Harvard Medical School, Boston, MA, United States; ^5^Department of Sound, Faculty of Arts, University of Chile, Santiago, Chile

**Keywords:** ambulatory monitoring, neck-surface accelerometer, subglottal pressure estimation, voice production model, neural networks, clinical voice assessment

## Abstract

The ambulatory assessment of vocal function can be significantly enhanced by having access to physiologically based features that describe underlying pathophysiological mechanisms in individuals with voice disorders. This type of enhancement can improve methods for the prevention, diagnosis, and treatment of behaviorally based voice disorders. Unfortunately, the direct measurement of important vocal features such as subglottal pressure, vocal fold collision pressure, and laryngeal muscle activation is impractical in laboratory and ambulatory settings. In this study, we introduce a method to estimate these features during phonation from a neck-surface vibration signal through a framework that integrates a physiologically relevant model of voice production and machine learning tools. The signal from a neck-surface accelerometer is first processed using subglottal impedance-based inverse filtering to yield an estimate of the unsteady glottal airflow. Seven aerodynamic and acoustic features are extracted from the neck surface accelerometer and an optional microphone signal. A neural network architecture is selected to provide a mapping between the seven input features and subglottal pressure, vocal fold collision pressure, and cricothyroid and thyroarytenoid muscle activation. This non-linear mapping is trained solely with 13,000 Monte Carlo simulations of a voice production model that utilizes a symmetric triangular body-cover model of the vocal folds. The performance of the method was compared against laboratory data from synchronous recordings of oral airflow, intraoral pressure, microphone, and neck-surface vibration in 79 vocally healthy female participants uttering consecutive /pæ/ syllable strings at comfortable, loud, and soft levels. The mean absolute error and root-mean-square error for estimating the mean subglottal pressure were 191 Pa (1.95 cm H_2_O) and 243 Pa (2.48 cm H_2_O), respectively, which are comparable with previous studies but with the key advantage of not requiring subject-specific training and yielding more output measures. The validation of vocal fold collision pressure and laryngeal muscle activation was performed with synthetic values as reference. These initial results provide valuable insight for further vocal fold model refinement and constitute a proof of concept that the proposed machine learning method is a feasible option for providing physiologically relevant measures for laboratory and ambulatory assessment of vocal function.

## 1. Introduction

Laryngeal voice disorders have been estimated to affect approximately 30% of the adult population in the United States at some point in their lives (Bhattacharyya, 2014). Voice disorders can disrupt or preclude normal oral communication and thus have far-reaching social, professional, economic, and personal consequences for those affected. The most common voice disorders are associated with detrimental patterns of daily vocal behavior and voice use (often classified as vocal hyperfunction) for which there is limited understanding of the underlying etiological and pathophysiological mechanisms. The paucity of such information serves to hinder the effective prevention, diagnosis and treatment of these common voice disorders.

Ambulatory voice monitoring using a neck-placed accelerometer (ACC) provides the capability to quantitatively assess daily vocal function and has also been shown to have the potential to assist in modifying vocal behaviors via ambulatory biofeedback (Popolo et al., 2005; Hillman and Mehta, 2011; Mehta et al., 2012; Andreassen et al., 2017; Van Stan et al., 2017a). Numerous features have been extracted from the ambulatory recording of the ACC signal, including phonation duration, sound pressure level (SPL), fundamental frequency (*f*_*o*_) (Ghassemi et al., 2014), vocal vibration-dose measures (Titze et al., 2003; Titze and Hunter, 2015), spectral and cepstral measures (Mehta et al., 2015, 2019), and aerodynamic measures (Llico et al., 2015; Cortés et al., 2018). These measures have been used to differentiate the daily voice use of patients with vocal hyperfunction from matched controls (Ghassemi et al., 2014; Cortés et al., 2018; Van Stan et al., 2021) and to track changes related to surgical and voice therapy treatment of hyperfunctional voice disorders (Van Stan et al., 2017b, 2020). Current classification accuracy using these parameters is in the range of 0.7–0.85.

We argue that the extraction of additional physiological measures from ambulatory ACC recordings, such as subglottal pressure, vocal fold collision pressure, and laryngeal muscle activation, would provide critical additional insights into the etiologic and pathophysiological mechanisms that underlie hyperfunctional voice disorders and thus significantly enhance the capability to identify the detrimental daily patterns of vocal behavior associated with these disorders (Espinoza et al., 2017; Galindo et al., 2017; Hillman et al., 2020). There have been recent efforts to develop subject-specific representations that can capture such physiologically relevant measures (e.g., subglottal pressure, contact pressure, muscle activation, and material properties of the vocal folds) that are difficult to obtain directly (Deng et al., 2019; Hadwin et al., 2019; Alzamendi et al., 2020; Drioli and Foresti, 2020). These approaches take advantage of the physiological relevance of lumped and finite element models of voice production, which have been shown to be useful tools for the investigation, diagnosis, and treatment of voice disorders (Erath et al., 2013). The most recent *in vivo* approach uses a Bayesian framework to estimate lumped-element vocal fold model parameters to predict subglottal pressure, vocal fold collision pressure, and laryngeal muscle activation along with their corresponding confidence intervals from observations obtained in clinical recordings, i.e., high-speed videoendoscopy (HSV) and oral airflow (Alzamendi et al., 2020).

Direct application of Bayesian subject-specific estimation from the ACC signal remains unsolved. There are challenges associated with the current extended Kalman filter approach for processing ambulatory data and using the ACC as the solely observation that remain to be addressed, including the large computational cost for the volume of data to be processed, the need for data fusion from different recording sessions, the need for an online estimation of model covariance, and the incorporation of a time-domain neck skin model for the ACC sensor within the voice production model.

On the other hand, machine learning and artificial intelligence are becoming relevant tools in biomedical engineering, as they can provide accurate predictions and efficient implementations. Numerical models are attractive alternatives for training purposes, suitable representing a significant range of conditions and providing access to relevant measures that are difficult to obtain experimentally. Voice assessment is starting to make use of these modeling advantages, where machine learning methods have been trained using simulated data from physiological numerical models to predict clinical parameters of interest. This approach was utilized by Gómez et al. (2019) to predict subglottal pressure from HSV in excised porcine vocal folds and by Zhang (2020) to predict vocal fold (geometric and mechanical) properties and subglottal pressure from a microphone signal. No machine learning method trained with a voice production model has been devised for the ACC signal in a laboratory or ambulatory context.

Although there are ongoing efforts to address the challenges of the Bayesian framework for the ambulatory monitoring, we propose in this study a more direct solution for the estimation of ambulatory physiologically-based features from the ACC that uses machine learning and voice modeling tools. Thus, we propose a method to obtain a non-linear optimal mapping between ACC features and subglottal pressure, vocal fold collision pressure, and laryngeal muscle activation. We propose using the impedance based inverse filtering (IBIF) algorithm (Zañartu et al., 2013; Cortés et al., 2018), which yields an unsteady glottal airflow signal from the ACC signal, to provide aerodynamic features that are used as inputs to the non-linear mapping. At the same time, we propose using a neural network (NN) regression architecture trained from a physiologically relevant muscle-controlled voice synthesizer with a triangular body-cover vocal fold model (Alzamendi et al., 2019, 2021) that takes the aerodynamic features as input and provides subglottal pressure, collision pressure, and laryngeal muscle activation as output. Predictions obtained with this scheme are validated against numerical simulations and laboratory measurements of subglottal pressure. The contributions of this work are twofold: First, the proposed scheme provides access, for the first time, to various physiologically relevant model-based features from a neck-surface accelerometer signal. Then, the approach provides a comprehensive contrast of the selected voice production model against laboratory data.

## 2. Materials and Methods

**Figure 1:**
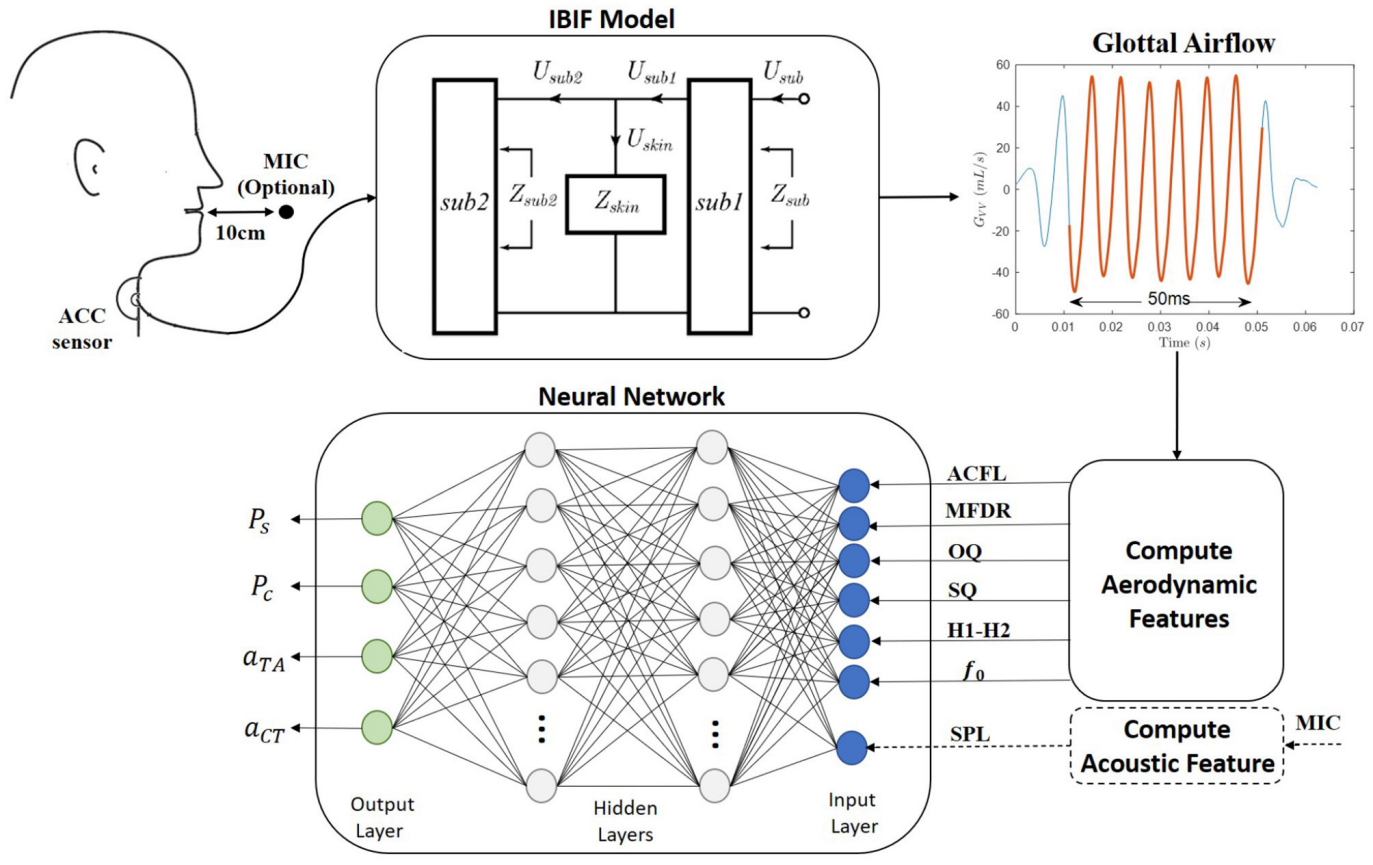
A schematic of the proposed method for the ambulatory vocal assessment based on processing the neck skin acceleration signal and a regression neural network.

Figure 1 provides an overall schematic of the proposed method of estimating four vocal function measures from neck-surface vibration recorded using a neck-surface accelerometer (ACC) sensor. The first analysis block results in an estimate of the unsteady glottal airflow volume velocity signal using the IBIF model (Zañartu et al., 2013), which has been shown to provide aerodynamic features reliably for the classification of vocal hyperfunction in laboratory (Espinoza et al., 2020) and ambulatory (Cortés et al., 2018) settings. The second analysis block computes the following six features from the glottal airflow signal: amplitude of the unsteady glottal airflow (ACFL), maximum flow declination rate (MFDR), open quotient (OQ), speed quotient (SQ), spectral tilt measured as the log-magnitude difference between the first and second harmonics (*H*_1_−*H*_2_), and fundamental frequency (*f*_*o*_). A seventh feature—the sound pressure level (SPL)—can be estimated either directly using an acoustic microphone (MIC) in the laboratory setting or using a log-log mapping between the root-mean-square magnitude of the ACC signal and SPL (Švec et al., 2005). See Table 1 for descriptions of each feature. These seven features are used as input into a NN to estimate four desirable measures of vocal function: subglottal pressure (*P*_*s*_), vocal fold collision pressure (*P*_*c*_), and normalized activation levels of the cricothyroid (*a*_*CT*_) and thyroarytenoid (*a*_*TA*_) muscles.

**Table 1:** Description of aerodynamic features extracted from the glottal airflow signal and acoustic sound pressure level extracted from the microphone or accelerometer signal.

**Feature**	**Description**	**Units**
ACFL	The difference between the maximum and minimum amplitude of the AC glottal airflow (peak-to-peak) within each glottal cycle	*mL*/*s*
MFDR	Maximum flow declination rate: Negative peak of the first derivative of the glottal waveform	*L*/*s*^2^
OQ	Open quotient: Ratio of the open time of the glottal vibratory cycle to the corresponding cycle period. Computed as in Cortés et al. (2018)	%
SQ	Speed quotient: Ratio of the opening time of the glottis to the closing time. Computed as in Cortés et al. (2018)	–
*H*_1_−*H*_2_	Difference between the magnitude of the first two harmonics	*dB*
*f*_*o*_	Fundamental frequency	*Hz*
SPL	Sound pressure level: dB from the RMS envelope of the acoustic signal	dB SPL

The NN was trained using 13,000 Monte Carlo simulations of a numerical voice production model. The design of the network architecture and overall training description are provided in section 2.1, and the details of the numerical voice production model are found in section 2.2. Validation of the estimated output features were performed using *in vivo* laboratory reference measures of *P*_*s*_ or numerical simulations of phonation for reference measures of *P*_*c*_, *a*_*CT*_, and *a*_*TA*_. Details of the experimental validation are provided in section 2.3.

### 2.1. Neural Network Architecture and Training

A supervised machine learning framework for regression was implemented based on a multi-layer NN (Hagan et al., 2014). The network consisted of an input layer of the seven aerodynamic and acoustic features (ACFL, MFDR, OQ, SQ, *H*_1_−*H*_2_, *f*_*o*_, and SPL), an output layer composed of the four target vocal function measures (*P*_*s*_, *P*_*c*_, *a*_*TA*_, and *a*_*CT*_), and two interconnected hidden layers with a 10% dropout to avoid overfitting. Each neuron within the hidden layers had adjustable weight and bias parameters that combined with the outputs of the preceding layer to activate a rectified linear unit activation function; then, the resulting activation served as input for the next layer Bianco et al. (2019). The number of neurons for each layer was investigated as a function of the model performance against both numerical and experimental data. The training stage updates the weights and biases using the Adam optimization algorithm (Kingma and Ba, 2017) with a learning rate of 0.001. All the NNs involved in this work were implemented in a virtual machine from Google Colaboratory with two CPU models Intel(R) Xeon(R) CPU @ 2.00GHz, using Python 3.7.11. and the TensorFlow 2.5.0 library (Abadi et al., 2015). The runtime for the largest network (8 hidden layer with 128 neurons and 100 epoch) was less than 120 s.

**Figure 2:**
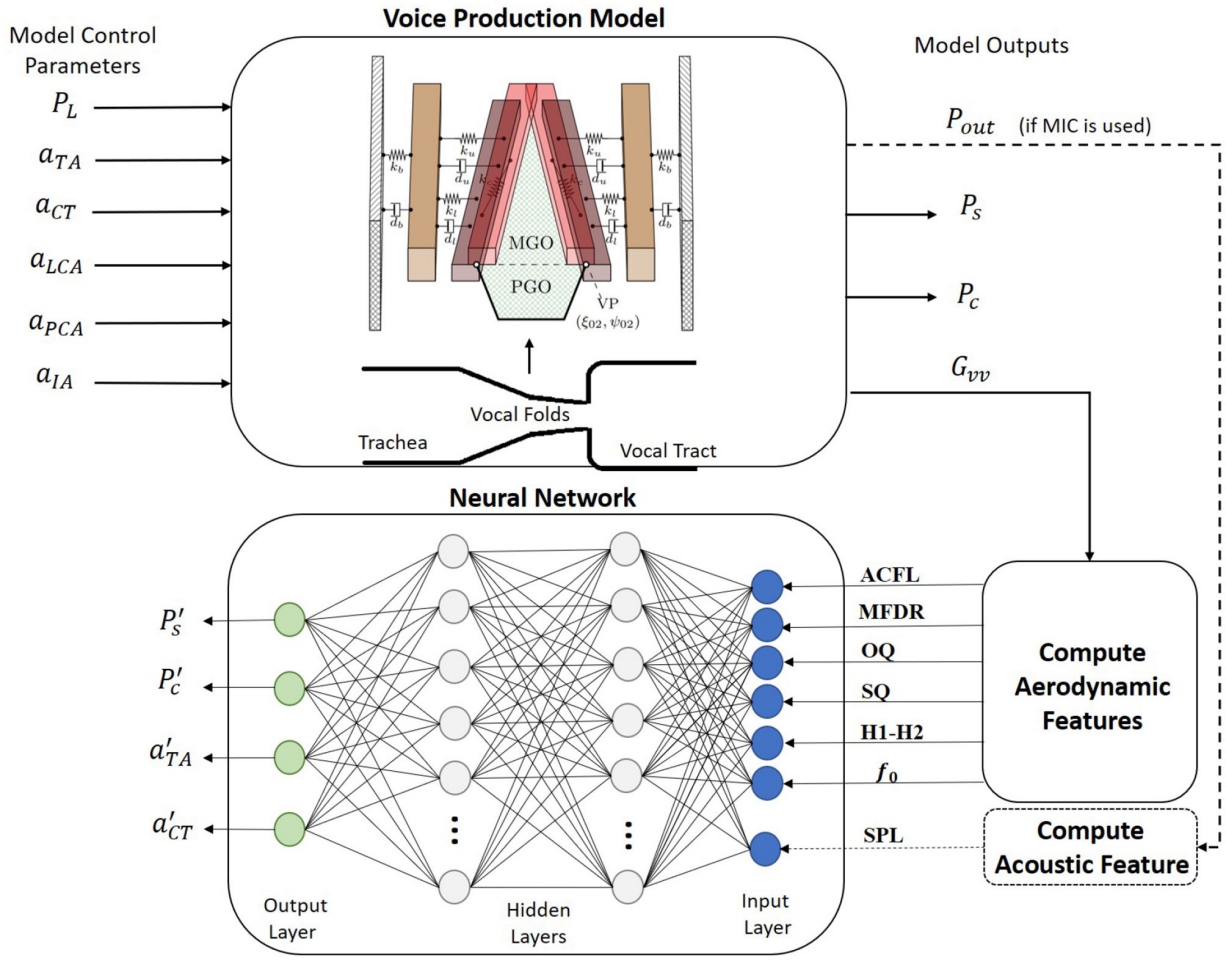
A schematic for the proposed training procedure. A regression neural network is built for mapping accelerometer-based vocal features into clinically relevant estimates for subglottal pressure, subglottal collision pressure, and laryngeal muscle activation levels of the thyroarytenoid (TA) and cricothyroid (CT) muscles. Training data are produced from a numerical voice production model.

The NN regression models were trained following the scheme shown in Figure 2. For this purpose, a synthetic voice dataset was obtained with a numerical voice production model described in section 2.2. Similar approaches were recently taken by other authors using different sensing modalities, i.e., high-speed videoendoscopy (Gómez et al., 2019) and MIC sensors (Zhang, 2020) in *ex vivo* experimental validation platforms (instead of *in vivo*). Using synthetic data for training helped addressing the lack of comprehensive and massive *in vivo* human datasets with thousands or even millions of conditions. Testing of the NN models was performed with both numerical and *in vivo* laboratory datasets. The laboratory dataset is described in section 2.3.

The voice production model described in section 2.2 was used to create 110,000 Monte Carlo simulations of sustained phonation. The simulations included a wide variation of the model control parameters such as lung pressure (*P*_*L*_), activation levels for the cricothyroid (*a*_*CT*_), thyroarytenoid (*a*_*TA*_), lateral cricoarytenoid (*a*_*LCA*_), interarytenoid (*a*_*IA*_), and posterior cricoarytenoid (*a*_*PCA*_) muscles. Control model parameters and their variation range are shown in Table 2. Each simulation lasted 800 ms, with the mean value of the seven input features taken for the last 50 ms to avoid transient artifacts. The glottal airflow was filtered using the same low- and high-pass filters utilized in the analysis of the laboratory recordings, as described in section 2.3.

**Table 2:** Range and increment step for control parameters in the numerical voice production model considered for building the synthetic dataset.

**Parameter**	**Range**	**Step**	**Unit**
*a*_*CT*_	0-1	0.1	–
*a*_*TA*_	0-1	0.1	–
*a*_*LCA*_	0.2-0.8	0.1	–
*a*_*PCA*_	0-0.1	0.1	–
*a*_*IA*_	0.2-0.8	0.1	–
*P*_*L*_	500 – 2000	150	Pa

**Figure 3:**
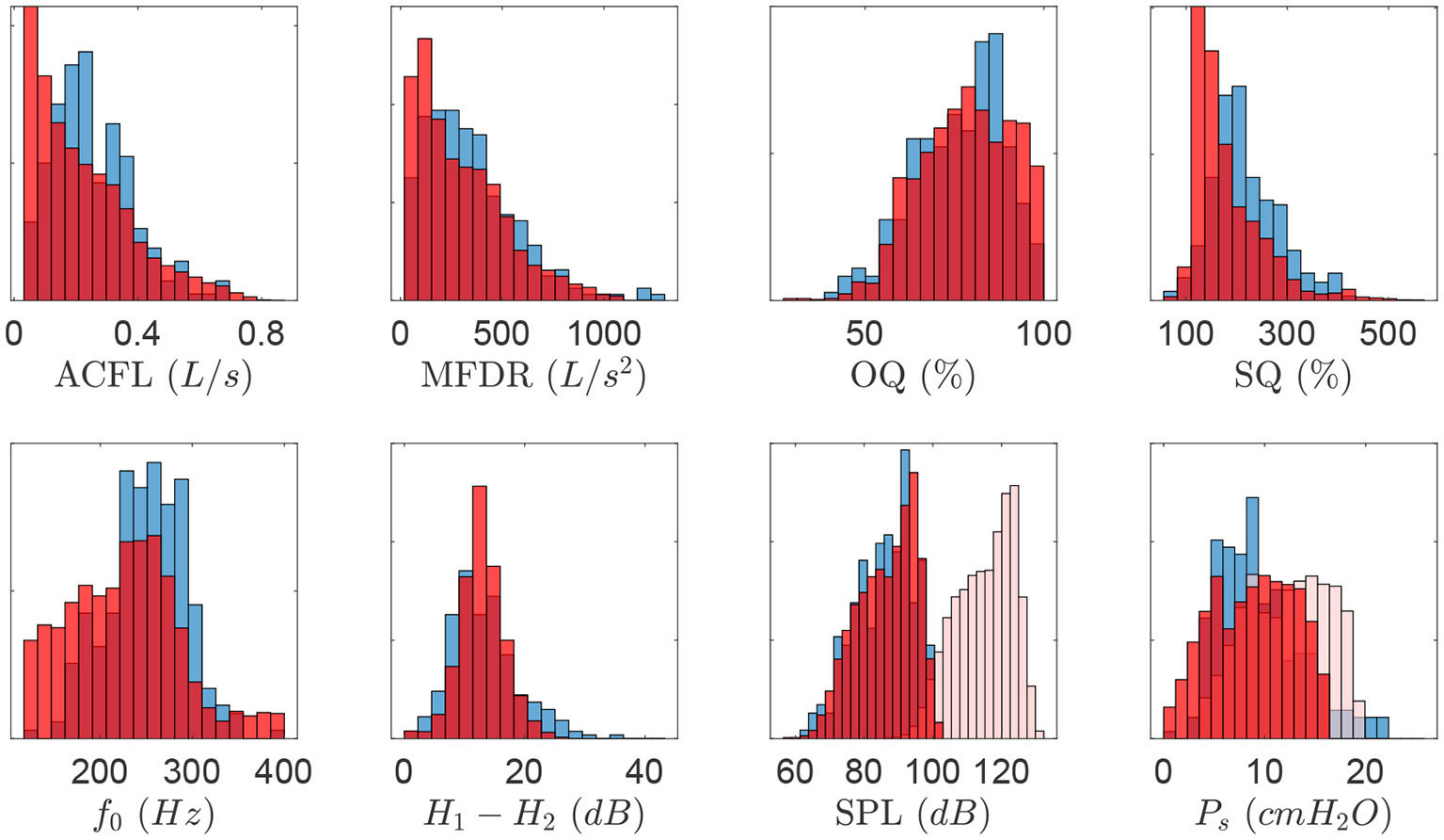
Normalized histogram describing vocal features obtained for all measured quantities in the clinical data set (blue color). Resulting histograms for the synthetic dataset (red color) are superimposed to illustrate model matching. Bias correction for synthetic SPL and Ps are shown as additional histograms (light red color).

As suggested by Gómez et al. (2019), the training data resembled the empirical distribution of the population-based aerodynamic and acoustic feature set. Thus, simulated data with ACFL less than 30 *mL*/*s* and *f*_*o*_ outside the range of 120–400 Hz were discarded, as these cases were not found in the laboratory data used for testing the NN. As a result, the final synthetic dataset consisted of 13,000 samples. Figure 3 shows the normalized histogram of features for the synthetic data (blue color) and laboratory data (red color).

Notice that feature ranges and distributions for both clinical and synthetic data sets agree, except for SPL and *P*_*s*_, where ranges are noticeable dissimilar (see histograms for attenuated red color). Two bias corrections were considered for these components. First, as the SPL for the voice production model is obtained at the lips, the SPL value was corrected to match the 10 cm mouth-to-microphone recording distance considered in the clinical recordings, yielding a −28.5 *dB* correction factor (Švec and Granqvist, 2018). In addition, histograms of *P*_*s*_ suggest that the physiological voice synthesizer yields higher values for this measure. It is possible that sub and supra glottal tract propagation losses and the losses at the glottal boundary were not sufficiently high, thus amplifying source-filter interactions and raising up subglottal pressure. This bias has motivated subsequent exploration and model developments. However, to address the need to correct for the difference in *P*_*s*_ in this study, a bias correction was applied by taking the differences between the mean of clinical and synthetic *P*_*s*_ values, thus leading to a −3.37 cm H_2_O offset.

Synthetic training data were min-max normalized and selected randomly from 80% of the total simulations. Testing was performed in the remaining 20% of synthetic data and in the clinical data in order to identify the models providing the best estimation of subglottal pressure. To assess the regression performance during both training and validation stages and to compare with prior studies (Gómez et al., 2018; Lin et al., 2020; Zhang, 2020), the mean absolute error (MAE) and the root-mean-squared error (RMSE) metrics were utilized.

Several NN architectures with different numbers of neurons in the hidden layers were trained for two cases. Case I included six glottal aerodynamic features, described in Table 1 (ACFL, MFDR, OQ, SQ, *f*_*o*_, and *H*_1_−*H*_2_) as input layer to the NNs, i.e., glottal measures extracted only from IBIF. Case II had the input layer of the NNs composed by all seven features in Table 1.

### 2.2. Voice Production Model

The selected voice production model for the training stage is a multi-physics scheme featuring a low-order model of the vocal folds that allows for the coordinated activation of all five intrinsic laryngeal muscles (Alzamendi et al., 2019, 2021). The model was recently developed and was chosen due to its flexibility and physical and physiological relevant way to cover numerous (normal and disordered) phonatory conditions. The approach builds upon prior efforts that describe rules for controlling low-order models (Titze and Story, 2002), vocal fold posturing (Titze and Hunter, 2007), and a triangular body-cover vocal fold model (Galindo et al., 2017). The model also accounts for tissue-fluid-acoustic interactions at the glottis (Zañartu et al., 2014), sound wave propagation through the vocal tract followed by sound pressure radiated from mouth (Zañartu, 2006), and allows for describing sustained vowels and time-varying glottal gestures. Given that the model is fairly new, we describe its main components pertaining to the development of the NN regression model and training set.

The triangular body-cover model (TBCM) (Galindo et al., 2017) (see Figure 2) consists of paired three-mass body-cover systems interconnected with mechanical elements (Story and Titze, 1995) and configured in a triangular anatomical shape (Birkholz et al., 2011). Beside resembling the triangular glottis, the TBCM is physiologically relevant because it mimics the layered vocal fold structure and extends the vocal fold collision model with a gradual zipper-like incomplete glottal closure. The latter aided to describe the time-varying vocal fold collision pressure (*P*_*c*_) during phonation. Similar to Galindo et al. (2017), the parameterization of the TBCM followed the original body-cover description (Story and Titze, 1995) and applied the empirical rules to change geometrical and viscoelastic vocal fold parameters developed by Titze and Story (2002). However, the major difference with (Galindo et al., 2017) resided in the computation of both the internal tension and elongation in the vocal folds. The remaining rules in Titze and Story (2002) were taken as originally proposed for deriving the lumped-element dynamical parameters.

Given the interest in estimating intrinsic laryngeal activity with the proposed method, a comprehensive description of muscle activity on the laryngeal configuration was considered. For this purpose, the contributions of all five intrinsic muscles and the passive response of connective tissue (i.e., the vocal ligament and vocal fold mucosa) were included in the model. Hence, simulated laryngeal muscle activations were the control variables governing the phonatory posture and vocal fold elongation. The incorporation of this muscle-controlled model of the larynx allowed to dynamically modify the glottal function during phonation, e.g., the vocal fold oscillatory dynamics, time-varying glottal resistance, and aerodynamic-acoustic coupling mechanisms. Following Titze and Hunter (2007), the five intrinsic muscles were modeled independently by using a modified Kelvin model (Hunter et al., 2004), which dynamically solves for the internal stress-strain response in one-dimensional fibrous tissues by integrating both active and passive properties. Passive stress was described as a non-linear function of longitudinal strain. Additionally, the active stress resulted from the maximum isometric active stress and the normalized activation level, in the range 0 ≤ *a* ≤ 1, mapping from relaxed to strong muscle tension. The simulated muscle activation for each intrinsic muscle was thus adjusted trough the corresponding activation levels {*a*_LCA_, *a*_IA_, *a*_PCA_, *a*_CT_, *a*_TA_}. In the TBCM, an adducted glottal configuration is critical for setting the system into self-sustained oscillations, thus requiring higher activation of the adductory intrinsic (LCA and IA) musculature than the abductury intrinsic (PCA) musculature. For simplicity, we did not consider the effects of elevated antagonistic muscles (Alzamendi et al., 2021) and only explored a small range of PCA activation to secure self-sustained oscillations in the TBCM. This approach allowed us to reduce the number of simulations to be discarded and to optimize the computational load. Future investigations will involve further scenarios for muscle control in typical and disordered phonation. Models for the vocal ligament and vocal fold mucosa were similarly implemented, except that the active component was set to zero for these cases (Titze, 2006).

Beside controlling intrinsic muscle activation, the voice production model also allowed for the adjustment of the aerodynamic lung pressure, *P*_*L*_. The aerodynamic forces acting over the vocal fold cover layer were then computed from the resulting subglottal pressure, *P*_*s*_, and supraglottal pressure, *P*_*e*_, according to Titze (2002). The three-way interaction at the glottal level between sound, flow, and vocal fold tissue was included, whereas the glottal airflow was computed from the acoustic driving pressures impinging on the glottal (membranous plus posterior portions) area following (Titze, 2006; Zañartu et al., 2014; Lucero and Schoentgen, 2015). Simulation of the time-varying acoustic wave propagation was achieved by applying the wave reflection analog scheme, where the subglottal and supraglottal tracts were modeled as a discrete concatenation of short uniform acoustic cylinders with variable cross-sectional areas. Effects due to the boundary condition at the lips was approximated by including an inertive radiation impedance, that produces the reflected pressure wave and the radiated sound wave, *P*_*out*_. Losses due to viscosity, moving walls, and other losses are described by an exponential attenuation factor in the propagation through the cylindrical sections (Zañartu, 2006; Zañartu et al., 2007). Vocal tract area functions that resemble a typical male (Story, 2008) and female (Story et al., 1998) that could match the *in vivo* experimental data were selected, i.e., vowels /æ/ and /ɑ, along with a representative subglottal tract (Zañartu et al., 2014).

### 2.3. Experimental Validation of NN-Estimated Subglottal Pressure

An *in vivo* laboratory dataset (Mehta et al., 2015; Espinoza et al., 2017, 2020) with synchronous recordings of intraoral pressure (IOP), oral airflow volume velocity (OVV), MIC, and ACC from vocally healthy subjects was utilized to provide a completely separate testing platform for the estimates obtained with the regression NN. This dataset was used to experimentally validate the NN estimates of subglottal pressure. Direct measurements of vocal fold collision pressure and laryngeal muscle activation are difficult to obtain in the laboratory and were not included in this experimental validation. Note that this dataset was not used to train the NN.

The data correspond to a group of participants composed of 79 adult females with no history of voice disorders. The mean (SD) age was 29.6 (13.0) years old. Their vocally healthy status was verified by a licensed speech-language pathologist via interview (subjects reported no difficulties with their voices in daily life), laryngeal videostroboscopic examination, and a clinician-administered Consensus Auditory-Perceptual Evaluation of Voice (CAPE-V) assessment (Kempster et al., 2009). Informed consent was obtained from all the participants in this study, and experimental and clinical protocols were approved by the institutional review board of Mass General Brigham (formerly Partners HealthCare) at the Massachusetts General Hospital. Data recordings were conducted in a sound-treated room where study staff instructed each participant to repeat strings of /pæ/ syllables in three loudness conditions (comfortable, loud, and soft). Although subjects were instructed to maintain a constant pitch and loudness within each syllable string, they were free to choose levels that were most natural for them without any prescribed levels of absolute pitch and loudness.

Recordings consisted of the simultaneous acquisition of acoustic pressure obtained with a condenser MIC (MKE104, Sennheiser, Electronic GmbH, Wedemark, Germany) placed 10 cm from the lips and having full bandwidth in the range of 0–6 kHz, OVV sensed by using a circumferentially vented pneumotachograph mask (PT-2E, Glottal Enterprises, Syracuse, NY) with a bandwidth of approximately 1.1 kHz, IOP measured with an oral catheter passed between the lips and connected to a low-bandwidth pressure sensor with an effective bandwidth of approximately 80 Hz (Espinoza et al., 2017), and ACC (BU-27135; Knowles Corp., Itasca, IL, USA) placed on the anterior neck surface halfway between the thyroid prominence and the suprasternal notch (Zañartu et al., 2013). All signals were sampled at 20 kHz/16 bits (Digidata 1440A, Axon Instruments, Inc.), low-pass filtered at 8-kHz cutoff frequency (CyberAmp Model 380, Axon Instruments, Inc.), and calibrated to physical units (Espinoza et al., 2017).

Signals obtained from the ACC and pneumotachograph mask were low-pass filtered at 1,100 Hz with a 10th-order Chebyshev Type II filter and decimated to 8,192 Hz. Then, a fourth-order Butterworth high-pass filter with cutoff frequency at 60 Hz was used to remove low-frequency components. The IOP signal was low-pass filtered at 80 Hz with a fifth-order Butterworth filter and then decimated to 256 Hz sample rate. All filters were applied with phase removal to avoid phase distortion (Perkell et al., 1994).

Reference values for subglottal pressure were obtained from IOP signals following (Espinoza et al., 2017). Driving pressure was extrapolated as the mean value of the two consecutive IOP plateaus produced by the combined lip closure and glottis opening prior to the /p/ sounds, that produced just before and after each vowel segment. The three middle syllables in each /pæ/ string were selected for the analysis, so that the initial and final portions were disregarded to avoid any evident transient dynamics. The estimated subglottal pressure was the average of these three-syllable values. Three reference measures per participant for comfortable, loud, and soft loudness conditions were obtained. Thus, a total of 237 /pæ/ tokens were used in this study.

The OVV-based glottal airflow was obtained through a common inverse filtering technique based on a single-notch filter with a conjugate pair of zeros and unity gain at DC at first vocal tract resonance (Perkell et al., 1991; Cheyne, 2006). Each single-notch filter was applied to a 50 ms stable portion of the middle /pæ/ string. The center frequency of the filter was determined following an optimization procedure developed by Espinoza et al. (2017).

The ACC-based glottal airflow was estimated using the IBIF scheme (Zañartu et al., 2013; Cortés et al., 2018). This method uses an acoustic transmission line model and a calibration step to obtain a set of subject-specific parameters corresponding to the neck-skin surface, length of the trachea, and accelerometer position (Zañartu, 2010; Zañartu et al., 2013; Cortés et al., 2018). These parameters are determined by minimizing the waveform error between the OVV-based glottal airflow (reference signal described previously) and the inverse filtered neck-skin ACC signal via a particle swarm optimization Kennedy and Eberhart (1995). The middle 50 ms of the glottal airflow signal estimated from IBIF was selected to compute the six acceleration-based aerodynamic feature (see Table 1). Even though SPL can be computed from the ACC signal using regression methods Švec et al. (2005), the synchronous microphone signal was used in this study to avoid introducing any additional estimation error at this point. Future work can be devoted to enhance current linear mapping between ACC and SPL.

Validation with human data is the gold standard to assess the ability of the NN regression scheme to represent *in vivo* data; but direct measurement of certain physiological measures of vocal function is not feasible. An advantage of using a voice production model to train a neural network is that we can estimate vocal function measures that are difficult to measure in practice, which is the case for vocal fold collision pressure and intrinsic muscle activation. Thus, the assessment of the estimates of subglottal pressure is described in terms of test sets from numerical simulations and laboratory data, whereas the estimates of vocal fold collision pressure and laryngeal muscle activation are only evaluated using a synthetic data test set.

## 3. Results

### 3.1. Subglottal Pressure Estimation

The MAE and RMSE describing *P*_*s*_ estimates for the different architectures are reported in Table 3 for both synthetic and clinical test data. For both cases I and II, additional hidden layers and neurons per layer yielded an improvement in subglottal pressure estimation when tested against the synthetic data. For example, in case I, MAE decreased from 1.98 cm H_2_O to 0.93 cm H_2_O from the simplest (2 hidden layers with 4 neurons) to a more complex (4 hidden layers with 128 neurons) architecture, respectively. In case II, MAE decreased from 1.84 cm H_2_O to 0.78 cm H_2_O for the same prior complexity in the NN architecture. This represents a reduction of more than 50% in MAE in both cases. A similar trend is observed with RMSE. An explanation for the improvement comes from the fact that the training and testing data were obtained from the same voice production model. Therefore, more complex NN models appear to capture efficiently the non-linear mechanisms of the model, which has been suggested by Zhang (2020), when training and testing with synthetic data from the same model. However, for the NN architectures composed over the six hidden layer with 128 neurons, the MAE and RMSE for synthetic data increase, showing that a deeper NN does not improve the estimation of subglottal pressure in this context.

**Table 3:** MAE and RMSE between the estimated *P*_*s*_ with the proposed NN regression model and the reference measures from synthetic and laboratory test data.

**Neurons in**	**Number of**	**Synthetic Data**		**Laboratory Data**	
**each hidden**	**hidden layers**	**MAE**	**RMSE**	**MAE**	**RMSE**
**layer**		**(cm H_**2**_O)**	**(cm H_**2**_O)**	**(cm H_**2**_O)**	**(cm H_**2**_O)**
**Case I:**					
4	2	1.98	2.51	2.23	2.82
8	2	1.81	2.34	2.28	2.86
16	2	1.35	1.83	2.56	3.13
32	2	1.18	1.64	2.82	3.43
64	2	1.02	1.48	2.89	3.50
128	2	0.99	1.68	2.94	3.58
128	4	0.93	1.33	3.17	3.87
128	6	0.97	1.38	3.14	3.85
128	8	1.01	1.45	3.12	3.76
**Case II:**					
4	2	1.84	2.42	1.95	2.48
8	2	1.87	2.43	1.97	2.52
16	2	1.27	1.74	2.42	2.98
32	2	1.13	1.58	2.55	3.17
64	2	0.99	1.42	2.88	3.45
128	2	0.90	1.30	2.98	3.58
128	4	0.78	1.12	3.23	3.87
128	6	0.87	1.21	3.04	3.71
128	8	1.00	1.38	3.08	3.70

*Errors are reported for different NN architecture (different number of neurons and hidden layers). Case I: Input aerodynamic features of ACFL, MFDR, OQ, SQ, f_o_, and H_1_−H_2_. Case II: Input aerodynamic features in Case I and acoustic SPL*.

**Figure 4:**
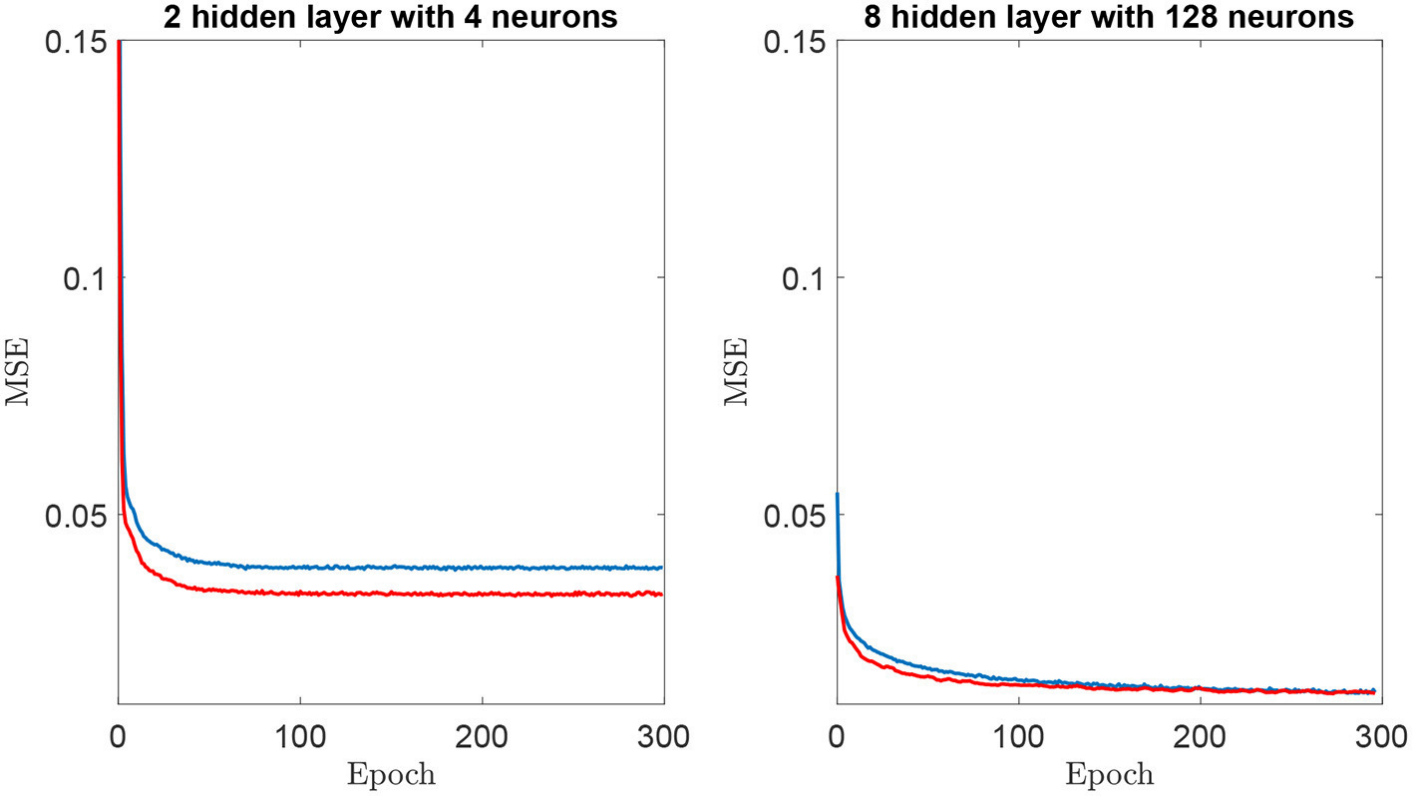
Mean Squared Error (MSE) vs. epoch for training (blue color) and validation (red color) for two neural networks architectures. **(Left)** 2 hidden layers with 4 neurons. **(Right)** 8 hidden layers and 128 neurons.

It is important to highlight that all NNs were trained using 100 epochs. This criterion was selected to ensure the convergence of models. Figure 4 shows mean squared error vs. the epochs for training and validation of the simplest and the most complex architecture models. The curves illustrate the convergence of the training procedure, where the simplest regression model exhibits a more rapid convergence. However, at around 100 epochs, the error remains constant, as the training progresses for both architectures. A similar trend was observed for all tested configurations. Another element to highlight is the absence of overfitting, since the training and validation error monotonically decrease at the same time. This shows that the network learns the structure of the observed data and is able to infer the validation data. An indication of overfitting would be a training error that decreases while the validation error remains the same or increases.

On the other hand, for the laboratory validation of subglottal pressure, we found the opposite trend for MAE as a function of the NN architecture complexity. In Case I, MAE increased from 2.23 cm H_2_O to 3.17 cm H_2_O for an increasing complexity from the 2 hidden layers with 4 neurons to 4 hidden layers with 128 neurons model. Case II also exhibited MAE increases from 1.95 cm H_2_O to 3.23 cm H_2_O for the same increasing complexity in the NN architecture. These results represented an increase of 42% and 66% in MAE for Case I and II, respectively, with similar trends for RMSE. Therefore, higher NN complexity was not adequate to represent sample distribution from the laboratory dataset.

Table 3 also illustrates that the inclusion of SPL in the input feature vector improves the estimation of subglottal pressure for all tested NN architectures. Using the best architecture for the laboratory validation, we found a 12% reduction in MAE and RMSE. The best architecture for the synthetic validation exhibited a 16% reduction in MAE and RMSE when SPL was added. These results are in agreement with previous studies (Titze et al., 2003; Björklund and Sundberg, 2016; Espinoza et al., 2017) that reported a strong correlation between subglottal pressure and acoustic SPL. Although not reported, no significant error differences were observed when estimating SPL from either the MIC or ACC sensor.

**Figure 5:**
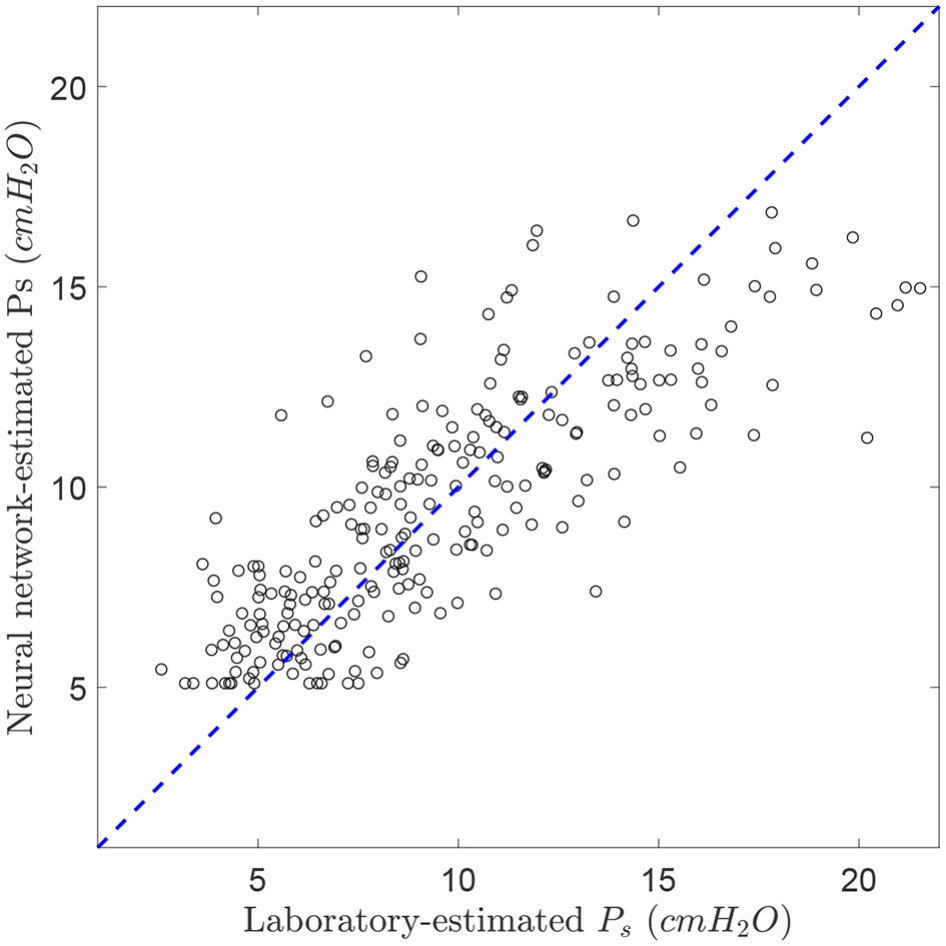
Comparison between laboratory-estimated subglottal pressure and the corresponding estimates from the trained neural network (2-hidden layer, 4 neurons in each layer, and 7 voice features). *R*^2^ = 0.65. The dashed line represents the theoretical 1:1 perfect matching.

Therefore, the NN model with lowest error in the validation set from the laboratory data was selected from 4 neurons in the hidden layers and all seven input features. Figure 5 shows a scatter plot of the NN-estimated subglottal pressure vs. the reference subglottal pressure from the laboratory data. The dashed line represents a 1:1 correspondence between the estimated and reference subglottal pressure. The coefficient of determination *R*^2^ is 0.65 and the mean absolute percentage error is 24.9%. We highlight that even though the IOP data was used as ground truth for this assessment, differences in the subglottal pressure estimates from IOP and direct measurement of subglottal pressure via tracheal puncture has been reported in the range of 5% (Hertegård et al., 1995), although interpolation between the peaks of the pulses can lead to a 12% error (Rothenberg, 2013).

### 3.2. Vocal Fold Collision Pressure and Laryngeal Muscle Activation Estimation

Table 4 reports the coefficient of determination *R*^2^, MAE (in physical units and in percentage of range) using synthetic data for the four outputs (*P*_*s*_, *P*_*c*_, *a*_*CT*_, *a*_*TA*_) obtained using the NN for the 2 hidden layers with 4-neuron and 4 the hidden layers with 128-neuron architectures. The architectures with more layers exhibited the best performance for estimating subglottal pressure for the synthetic data.

**Table 4:** Assessment of estimated vocal measures *P*_*s*_, *P*_*c*_, *a*_*TA*_, and *a*_*CT*_ using the proposed NN regression method.

**Parameters**	**Units**	***R*^2^**	**MAE**	**MAE**
			**(Units)**	**(%)**
**2-HL and 4-N architecture:**				
*P*_*s*_	cm H_2_O	0.64	1.84	11.4
*P*_*c*_	cm H_2_O	0.70	3.33	8.2
*a*_*TA*_	-	0.07	0.21	21.1
*a*_*CT*_	-	0.53	0.15	14.6
**4-HL and 128-N architecture:**				
*P*_*s*_	cm H_2_O	0.93	0.74	4.7
*P*_*c*_	cm H_2_O	0.92	1.70	4.2
*a*_*TA*_	–	0.52	0.13	13.3
*a*_*CT*_	–	0.84	0.07	7.1

*Reported values for R^2^ and MAE (in physical units and in percentage of range) for two NN architectures with different hidden layers (HL) and neurons (N). The input vector includes the seven aerodynamic measures*.

As seen before for the synthetic validation of subglottal pressure, increasing the complexity of the NN architecture increases the accuracy of the estimates. This performance holds true for the estimates of vocal fold collision pressure and laryngeal muscle activation. However, there is a significantly smaller *R*^2^ of 0.52 for *a*_*TA*_ estimation when compared with *R*^2^>0.8 for estimation of the other measures using the 4 hidden layer with 128 neurons NN. This finding suggests that certain measures, such as *a*_*TA*_, require deeper, more complex NN architectures to achieve similar performance.

## 4. Discussion

The purpose of this study was to explore the combination of neural network regression networks with a voice production model to estimate physiologically relevant vocal measures, i.e., subglottal pressure, vocal fold collision pressure, and (TA and CT) laryngeal muscle activation from a neck-surface vibration signal. Validation for this study was done both numerically and experimentally. Given that some of the predicted measures are difficult to obtain experimentally, only the estimates of subglottal pressure could be compared with reference estimates of mean subglottal pressure derived from the standard airflow interruption technique in the laboratory.

Both numerical and experimental validation experiments yielded reasonable accuracy. The robust and reliable estimates of the proposed method are dependent on the capacity of the selected voice production model to mimic the observed distributions in the laboratory data. As the architecture complexity of the NN increased, the estimation error decreased for the synthetic data but increases for the laboratory data. We argue that this is a result of the way the model was utilized, i.e., model parameters were swept across a large range of values, but no anatomical changes were considered; thus the model simply described a single subject for a range of conditions. This may be playing a role in the accuracy for the estimation of subglottal pressure because no inter-subject variability was considered. At the same time, we discarded cases that differed from the laboratory distributions during the training process and corrected for a bias in the estimation of subglottal pressure. In addition, it is possible that subglottal and supraglottal tract propagation losses and the losses at the glottal boundary were not high enough, thus amplifying the source-filter interactions and resulting subglottal pressure. Future efforts will be devoted to improve model development, better reflect population behaviors, and assess these effects in the predicted accuracy of the proposed approach. In spite of its simplicity and the aforementioned limitations, we still conclude that the triangular body cover model provides a good general representation of typical sustained phonation for a large range of subjects and conditions.

The predicted subglottal pressure in this study are comparable with those obtained in previous studies. Our lowest mean absolute error for estimated subglottal pressure from clinical data was 191 Pa (1.95 cm H_2_O), whereas two relevant studies reported mean absolute errors of 194 Pa (Gómez et al., 2019) and 115 Pa (Zhang, 2020). However, it is important to highlight that our predictions are obtained from a neck-surface accelerometer and that we tested our predictions against *in vivo* human data, whereas these studies used porcine and human excised larynx experiments. Lin et al. (2020) estimated subglottal pressure from a neck-surface accelerometer using a subject-specific step-wise factorial regression model in 26 normal subjects. The investigators obtained an average root-mean-square error in the range of 2.4–2.5 cm H_2_O, which is comparable with the root-mean-square error of 2.48 cm H_2_O in the current study. The linear regression models in Lin et al. (2020) included cepstral peak prominence and fundamental frequency, along with ACC-based aerodynamic measures and were constructed on an individual basis for every subject across multiple elicited voice qualities. The main advantages of the NN model approach are the fact that a single, general non-linear regression mapping is utilized and that our mapping also provides estimates of other clinically relevant measures of vocal function. It is acknowledged that future work is required to experimentally validate these other measures of vocal fold collision pressure and laryngeal muscle activation.

Titze et al. (2003) put forth a simple, empirically derived formula (Equation 15) that computed subglottal pressure using only measurements of SPL and *f*_*o*_. Applying this formula to the laboratory data (237 tokens) in the current study to estimate subglottal pressure resulted in a root-mean-square error of 2.86 cm H_2_O and mean absolute error of 2.11 cm H_2_O. The relatively good performance for such a simple formula supports the idea that simple regression architectures are adequate for predicting subglottal pressure in vocally typical conditions; estimation accuracy of linear regression models reduces when non-modal voice qualities are included (Marks et al., 2019, 2020; Lin et al., 2020). The model-based approach of the current work allows for the estimation of additional measures of vocal function (e.g., vocal fold collision pressure, laryngeal muscle activation).

On the other hand, the accuracy of estimates of muscle activation and collision pressure that was assessed against synthetic data was hampered by the simplicity of the rather shallow NN architecture that resulted from matching clinical data for subglottal pressure. When the complexity of the network is increased, the estimation of muscle activation and collision pressure improves. This result is encouraging to investigate the development of subject-specific models that can handle more complex neural network architectures without losing the ability to predict subglottal pressure.

These initial results constitute a proof of concept that the proposed NN method is a feasible option for estimating clinically relevant vocal measures that are difficult to directly measure in laboratory and ambulatory settings. Current results could be significantly improved by exploring different NN architectures, improving model development, training with ACC signal features directly (vs. model features), using subject-specific tuning with transfer learning instead of a generic training for all subjects, and including experimental validation of all predicted values. This study delineates a path for various subsequent research efforts in this direction.

The neck-surface accelerometer sensor can be worn by a speaker for laboratory, clinical, and ambulatory assessments of vocal function. The estimation of subglottal pressure was validated using sustained phonation datasets from numerical modeling and laboratory recordings. There is potential to translate this method into ambulatory settings due to the input of the network only needing accelerometer-based features for short-time windows of 50 ms in duration. We hypothesize that the physiologically relevant measures that are obtained with the proposed approach will yield salient measures of vocal function in real-world environments. We expect that the physiologically relevant measures that are obtained with the proposed approach will provide unique quantitatively based insights into the etiologic and pathophysiological mechanisms associated with daily voice use in patients with hyperfunctional voice disorders. The capability to link model outputs with clinical data is expected to produce more comprehensive and specific descriptions of aberrant phonatory mechanisms that will lead to better subclassification (phenotyping) of hyperfunctional voice disorders and ultimately improve the prevention, diagnosis, and treatment of these disorders.

## 5. Conclusion

A framework to estimate subglottal pressure, collision pressure, and muscle activation from a neck surface accelerometer is developed integrating machine learning tools and a numerical model of voice production. Aerodynamic measures estimated from the neck surface accelerometer are combined with a sound pressure level estimate obtained from either an accelerometer or a microphone, and are selected as inputs to a neural regression network. The non-linear mapping is trained solely with a low-order voice production model featuring a symmetric triangular body-cover model of the vocal folds. When compared with clinical recordings from 79 female vocally healthy participants, the mean absolute error and root mean square error for the subglottal pressure were 1.95 cm H_2_O and 2.48 cm H_2_O. These results are comparable with previous studies but with the advantage of having a general mapping for all patients and providing simultaneous estimates of collision pressure and muscle activation. However, given that clinical validation for these latter features is cumbersome, only synthetic data were used for that purpose, and experimental validation is left for future efforts. At the same time, relevant insights are gained by comparing the numerical model with the clinical data that will lead to further model refinements. The initial results constitute a proof of concept that the proposed machine learning method is a feasible option for providing highly relevant physical measures for the ambulatory assessment of voice. Future efforts will be focused on creating individualized mappings for normal and disordered voices with transfer learning and validating all the estimated features with *in vivo* recordings.

## Data Availability Statement

The datasets presented in this study can be found in online repositories. The names of the repository/repositories and accession number(s) can be found in the article/supplementary material.

## Ethics Statement

The studies involving human participants were reviewed and approved by Institutional Review Board of Mass General Brigham. The patients/participants provided their written informed consent to participate in this study.

## Author Contributions

MZ conceived the study and contributed to data analysis and interpretation, supervision, and manuscript writing. EI and JP conducted numerical simulations, neural network design, training and testing, data analysis and interpretation, and manuscript writing. GA developed the triangular body-cover model and contributed to data analysis and interpretation, supervision, and manuscript writing. JC and VE contributed to data analysis and interpretation and manuscript writing. DM and RH contributed to laboratory data collection, results interpretation, clinical input, and review editing. All authors contributed to the article and approved submitted version.

## Author Disclaimer

The content is solely the responsibility of the authors and does not necessarily represent the official views of the National Institutes of Health.

## Conflict of Interest

MZ has a financial interest in Lanek SPA, a company focused on developing and commercializing biomedical devices and technologies. MZ's interests were reviewed and are managed by Universidad Técnica Federico Santa María in accordance with its conflict-of-interest-policies. RH and DM have a financial interest in InnoVoyce LLC, a company focused on developing and commercializing technologies for the prevention, diagnosis, and treatment of voice-related disorders. RH's and DM's interests were reviewed and are managed by Massachusetts General Hospital and Mass General Brigham in accordance with their conflict-of-interest policies. The remaining authors declare that the research was conducted in the absence of any commercial or financial relationships that could be construed as a potential conflict of interest.

## Publisher's Note

All claims expressed in this article are solely those of the authors and do not necessarily represent those of their affiliated organizations, or those of the publisher, the editors and the reviewers. Any product that may be evaluated in this article, or claim that may be made by its manufacturer, is not guaranteed or endorsed by the publisher.

## References

[B1] Abadi M. Agarwal A. Barham P. Brevdo E. Chen Z. Citro C.. (2015). TensorFlow: Large-Scale Machine Learning on Heterogeneous Systems. Available online at: tensorflow.org

[B2] Alzamendi G. Manríquez R. Hadwin P. Deng J. Peterson S. Erath B.. (2020). Bayesian estimation of vocal function measures using laryngeal high-speed videoendoscopy and glottal airflow estimates: an *in vivo* case study. J. Acoust. Soc. Am. 147, EL434–EL439. 10.1121/10.0001276 32486812 PMC7480079

[B3] Alzamendi G. Peterson S. Erath B. Zaartu M. (2019). “Updated rules for constructing a triangular body-cover model of the vocal folds from intrinsic laryngeal muscle activation,” in The 13th International Conference on Advances in Quantitative Laryngology, Voice and Speech Research (Montreal, QC).

[B4] Alzamendi G. A. Peterson S. D. Erath B. D. Hillman R. E. Zaartu M. (2021). Triangular body-cover model of the vocal folds with coordinated activation of five intrinsic laryngeal muscles with applications to vocal hyperfunction. arXiv preprint arXiv:2108.01115. 10.1121/10.0009169 PMC8727069 35105008

[B5] Andreassen M. L. Litts J. K. Randall D. R. (2017). Emerging techniques in assessment and treatment of muscle tension dysphonia. Curr. Opin. Otolaryngol. Head Neck Surg. 25, 447–452. 10.1097/MOO.0000000000000405 28984699

[B6] Bhattacharyya N. (2014). The prevalence of voice problems among adults in the united states. Laryngoscope 124, 2359–2362. 10.1002/lary.24740 24782443

[B7] Bianco M. J. Gerstoft P. Traer J. Ozanich E. Roch M. A. Gannot S.. (2019). Machine learning in acoustics: theory and applications. J. Acoust. Soc. Am. 146, 3590–3628. 10.1121/1.5133944 31795641

[B8] Birkholz P. Kröger B. J. Neuschaefer-Rube C. (2011). “Synthesis of breathy, normal, and pressed phonation using a two-mass model with a triangular glottis,” in Interspeech 2011: 12th Annual Conference ofthe International Speech Communi- cation Association (Florence), 2681–2684.

[B9] Björklund S. Sundberg J. (2016). Relationship between subglottal pressure and sound pressure level in untrained voices. J. Voice 30, 15–20. 10.1016/j.jvoice.2015.03.006 25913752

[B10] Cheyne H. A. (2006). “Estimating glottal voicing source characteristics by measuring and modeling the acceleration of the skin on the neck,” in 2006 3rd IEEE/EMBS International Summer School on Medical Devices and Biosensors, Boston, MA, 118–121.

[B11] Cortés J. P. Espinoza V. M. Ghassemi M. Mehta D. D. Van Stan J. H. Hillman R. E.. (2018). Ambulatory assessment of phonotraumatic vocal hyperfunction using glottal airflow measures estimated from neck-surface acceleration. PLoS ONE 13:e0209017. 10.1371/journal.pone.0209017 30571719 PMC6301575

[B12] Deng J. J. Hadwin P. J. Peterson S. D. (2019). The effect of high-speed videoendoscopy configuration on reduced-order model parameter estimates by bayesian inference. J. Acoust. Soc. Am. 146, 1492–1502. 10.1121/1.5124256 31472542 PMC6715443

[B13] Drioli C. Foresti G. L. (2020). Fitting a biomechanical model of the folds to high-speed video data through bayesian estimation. Inform. Med. Unlocked 20:100373. 10.1016/j.imu.2020.100373

[B14] Erath B. D. Zañartu M. Stewart K. C. Plesniak M. W. Sommer D. E. Peterson S. D. (2013). A review of lumped-element models of voiced speech. Speech Commun. 55, 667–690. 10.1016/j.specom.2013.02.002

[B15] Espinoza V. M. Mehta D. D. Stan J. H. V. Hillman R. E. Zañartu M. (2020). Glottal aerodynamics estimated from neck-surface vibration in women with phonotraumatic and nonphonotraumatic vocal hyperfunction. J. Speech Lang. Hear. Res. 63, 2861–2869. 10.1044/2020_JSLHR-20-00189 32755502 PMC7890221

[B16] Espinoza V. M. Zañartu M. Stan J. H. V. Mehta D. D. Hillman R. E. (2017). Glottal aerodynamic measures in women with phonotraumatic and nonphonotraumatic vocal hyperfunction. J. Speech Lang. Hear Res. 60, 2159–2169. 10.1044/2017_JSLHR-S-16-0337 28785762 PMC5829799

[B17] Galindo G. E. Peterson S. D. Erath B. D. Castro C. Hillman R. E. Zañartu M. (2017). Modeling the pathophysiology of phonotraumatic vocal hyperfunction with a triangular glottal model of the vocal folds. J. Speech Lang. Hear. Res. 60, 2452–2471. 10.1044/2017_JSLHR-S-16-0412 28837719 PMC5831616

[B18] Ghassemi M. Van Stan J. H. Mehta D. D. Zañartu M. Cheyne H. A. II. Hillman R. E.. (2014). Learning to detect vocal hyperfunction from ambulatory neck-surface acceleration features: initial results for vocal fold nodules. IEEE Trans. Biomed. Eng. 61, 1668–1675. 10.1109/TBME.2013.2297372 24845276 PMC4077201

[B19] Gómez P. Schützenberger A. Kniesburges S. Bohr C. Dllinger M. (2018). Physical parameter estimation from porcine *ex vivo* vocal fold dynamics in an inverse problem framework. Biomech. Model Mechanobiol. 17, 777–792. 10.1007/s10237-017-0992-5 29230589

[B20] Gómez P. Schützenberger A. Semmler M. Döllinger M. (2019). Laryngeal pressure estimation with a recurrent neural network. IEEE J. Transl. Eng. Health Med. 7, 1–11. 10.1109/JTEHM.2018.2886021 30680252 PMC6331197

[B21] Hadwin P. J. Motie-Shirazi M. Erath B. D. Peterson S. D. (2019). Bayesian inference of vocal fold material properties from glottal area waveforms using a 2D finite element model. Appl. Sci. 9:2735. 10.3390/app9132735 34046213 PMC8153513

[B22] Hagan M. Demuth H. Beale M. De Jesús O. (2014). Neural Network Design. Stillwater, OK: Martin Hagan.

[B23] Hertegård S. Gauffin J. Åke Lindestad P. (1995). A comparison of subglottal and intraoral pressure measurements during phonation. J. Voice 9, 149–155. 7620537 10.1016/s0892-1997(05)80248-6

[B24] Hillman R. E. Mehta D. D. (2011). Ambulatory monitoring of daily voice use. Perspect. Voice Disord. 21, 56–61. 10.1044/vvd21.2.56

[B25] Hillman R. E. Stepp C. E. Stan J. H. V. Zañartu M. Mehta D. D. (2020). An updated theoretical framework for vocal hyperfunction. Am. J. Speech Lang. Pathol. 29, 2254–2260. 10.1044/2020_AJSLP-20-00104 33007164 PMC8740570

[B26] Hunter E. J. Titze I. R. Alipour F. (2004). A three-dimensional model of vocal fold abduction/adduction. J. Acoust. Soc. Am. 115, 1747–1759. 10.1121/1.1652033 15101653 PMC1550351

[B27] Kempster G. B. Gerratt B. R. Abbott K. V. Barkmeier-Kraemer J. Hillman R. E. (2009). Consensus auditory-perceptual evaluation of voice: development of a standardized clinical protocol. Am. J. Speech Lang. Pathol. 18, 124–132. 10.1044/1058-0360(2008/08-0017) 18930908

[B28] Kennedy J. Eberhart R. C. (1995). “Particle swarm optimization,” in Proceedings of the IEEE International Conference on Neural Networks, Perth, WA, 1942–1948.

[B29] Kingma D. P. Ba J. (2017). Adam: a method for stochastic optimization. arXiv preprint arXiv:1412.6980.

[B30] Lin J. Z. Espinoza V. M. Marks K. L. Zañartu M. Mehta D. D. (2020). Improved subglottal pressure estimation from neck-surface vibration in healthy speakers producing non-modal phonation. IEEE J. Select. Top. Signal Process. 14, 449–460. 10.1109/jstsp.2019.2959267 34079612 PMC8168553

[B31] Llico A. F. Zañartu M. Gonzalez A. J. Wodicka G. R. Mehta D. D. Van Stan J. H.. (2015). Real-time estimation of aerodynamic features for ambulatory voice biofeedback. J. Acoust. Soc. Am. 138, EL14–EL19. 10.1121/1.4922364 26233054 PMC4499052

[B32] Lucero J. C. Schoentgen J. (2015). Smoothness of an equation for the glottal flow rate versus the glottal area. J. Acoust. Soc. Am. 137, 2970–2973. 10.1121/1.4919297 25994724

[B33] Marks K. L. Lin J. Z. Burns J. A. Hron T. A. Hillman R. E. Mehta D. D. (2020). Estimation of subglottal pressure from neck surface vibration in patients with voice disorders. J. Speech Lang. Hear. Res. 63, 2202–2218. 10.1044/2020_JSLHR-19-00409 32610028 PMC7838842

[B34] Marks K. L. Lin J. Z. Fox A. B. Toles L. E. Mehta D. D. (2019). Impact of nonmodal phonation on estimates of subglottal pressure from neck-surface acceleration in healthy speakers. J. Speech Lang. Hear. Res. 62, 3339–3358. 10.1044/2019_JSLHR-S-19-0067 31518510 PMC6808343

[B35] Mehta D. D. Espinoza V. M. Van Stan J. Zañartu M. Hillman R. (2019). The difference between first and second harmonic amplitudes correlates between glottal airflow and neck-surface accelerometer signals during phonation. J. Acoust. Soc. Am. 145, EL386–EL392. 10.1121/1.5100909 31153299 PMC6520097

[B36] Mehta D. D. Van Stan J. H. Zañartu M. Ghassemi M. Guttag J. V. Espinoza V. M.. (2015). Using ambulatory voice monitoring to investigate common voice disorders: research update. Front. Bioeng. Biotechnol. 3:155. 10.3389/fbioe.2015.00155 26528472 PMC4607864

[B37] Mehta D. D. Zañartu M. Feng S. W. Cheyne H. A. II. Hillman R. E. (2012). Mobile voice health monitoring using a wearable accelerometer sensor and a smartphone platform. IEEE Trans. Biomed. Eng. 59, 3090–3096. 10.1109/TBME.2012.2207896 22875236 PMC3539821

[B38] Perkell J. S. Hillman R. E. Holmberg E. B. (1994). Group differences in measures of voice production and revised values of maximum airflow declination rate. J. Acoust. Soc. Am. 96, 695–698. 10.1121/1.410307 7930069

[B39] Perkell J. S. Holmberg E. B. Hillman R. E. (1991). A system for signal processing and data extraction from aerodynamic, acoustic, and electroglottographic signals in the study of voice production. J. Acoust. Soc. Am. 89, 1777–1781. 2045586 10.1121/1.401011

[B40] Popolo P. S. Svec J. G. Titze I. R. (2005). Adaptation of a pocket PC for use as a wearable voice dosimeter. J. Speech Lang. Hear. Res. 48, 780–791. 10.1044/1092-4388(2005/054) 16378473

[B41] Rothenberg M. (2013). “Rethinking the interpolation method for estimating subglottal pressure,” in Proceedings of the 10th International Conference on Advances in Quantitative Laryngology, Voice and Speech Research (Cincinnati, OH: AQL Press), 111–112.

[B42] Story B. H. (2008). Comparison of magnetic resonance imaging-based vocal tract area functions obtained from the same speaker in 1994 and 2002. J. Acoust. Soc. Am. 123, 327–335. 10.1121/1.2805683 18177162 PMC2377017

[B43] Story B. H. Titze I. R. (1995). Voice simulation with a body-cover model of the vocal folds. J. Acoust. Soc. Am. 97, 1249–1260. 7876446 10.1121/1.412234

[B44] Story B. H. Titze I. R. Hoffman E. A. (1998). Vocal tract area functions for an adult female speaker based on volumetric imaging. J. Acoust. Soc. Am. 104, 471–487. 9670539 10.1121/1.423298

[B45] Švec J. G. Granqvist S. (2018). Tutorial and guidelines on measurement of sound pressure level in voice and speech. J. Speech Lang. Hear. Res. 61, 441–461. 10.1044/2017_JSLHR-S-17-0095 29450495

[B46] Švec J. G. Titze I. R. Popolo P. S. (2005). Estimation of sound pressure levels of voiced speech from skin vibration of the neck. J. Acoust. Soc. Am. 117, 1386–1394. 10.1121/1.1850074 15807026

[B47] Titze I. R. (2002). Regulating glottal airflow in phonation: application of the maximum power transfer theorem to a low dimensional phonation model. J. Acoust. Soc. Am. 111, 367–376. 10.1121/1.1417526 11831809

[B48] Titze I. R. (2006). The Myoelastic Aerodynamic Theory of Phonation, 1st Edn. Iowa, IA: National Center for Voice and Speech.

[B49] Titze I. R. Hunter E. J. (2007). A two-dimensional biomechanical model of vocal fold posturing. J. Acoust. Soc. Am. 121, 2254–2260. 10.1121/1.2697573 17471739 PMC6371396

[B50] Titze I. R. Hunter E. J. (2015). Comparison of vocal vibration-dose measures for potential-damage risk criteria. J. Speech Lang. Hear. Res. 58, 1425–1439. 10.1044/2015_JSLHR-S-13-0128 26172434 PMC4686305

[B51] Titze I. R. Story B. H. (2002). Rules for controlling low-dimensional vocal fold models with muscle activation. J. Acoust. Soc. Am. 112(3 Pt 1), 1064–1074. 10.1121/1.1496080 12243155

[B52] Titze I. R. Svec J. G. Popolo P. S. (2003). Vocal dose measures: quantifying accumulated vibration exposure in vocal fold tissues. J Speech Lang. Hear. Res. 46, 919–932. 10.1044/1092-4388(2003/072) 12959470 PMC3158591

[B53] Van Stan J. H. Mehta D. D. Hillman R. E. (2017a). Recent innovations in voice assessment expected to impact the clinical management of voice disorders. Perspect. ASHA Spcl. Interest Groups 2, 4–13. 10.1044/persp2.SIG3.4

[B54] Van Stan J. H. Mehta D. D. Ortiz A. J. Burns J. A. Marks K. L. Toles L. E.. (2020). Changes in a daily phonotrauma index after laryngeal surgery and voice therapy: implications for the role of daily voice use in the etiology and pathophysiology of phonotraumatic vocal hyperfunction. J. Speech Lang. Hear. Res. 63, 3934–3944. 10.1044/2020_JSLHR-20-00168 33197360 PMC8608140

[B55] Van Stan J. H. Mehta D. D. Sternad D. Petit R. Hillman R. E. (2017b). Ambulatory voice biofeedback: relative frequency and summary feedback effects on performance and retention of reduced vocal intensity in the daily lives of participants with normal voices. J. Speech Lang. Hear. Res. 60, 853–864. 10.1044/2016_JSLHR-S-16-0164 28329366 PMC5548081

[B56] Van Stan J. H. Ortiz A. J. Cortes J. P. Marks K. L. Toles L. E. Mehta D. D.. (2021). Differences in daily voice use measures between female patients with nonphonotraumatic vocal hyperfunction and matched controls. J. Speech Lang. Hear. Res. 64, 1457–1470. 10.1044/2021_JSLHR-20-00538 33900807 PMC8608188

[B57] Zañartu M. (2006). Influence of acoustic loading on the flow-induced oscillations of single mass models of the human larynx (Master's thesis). School of Electrical and Computer Engineering, Purdue University, West Lafayette, IN, United States.

[B58] Zañartu M. (2010). Acoustic coupling in phonation and its effect on inverse filtering of oral airflow and neck surface acceleration (Ph.D. thesis). School of Electrical and Computer Engineering, Purdue University, West Lafayette, IN, United States.

[B59] Zañartu M. Galindo G. E. Erath B. D. Peterson S. D. Wodicka G. R. Hillman R. E. (2014). Modeling the effects of a posterior glottal opening on vocal fold dynamics with implications for vocal hyperfunction. J. Acoust. Soc. Am. 136, 3262–3271. 10.1121/1.4901714 25480072 PMC4257958

[B60] Zañartu M. Ho J. C. Mehta D. D. Hillman R. E. Wodicka G. R. (2013). Subglottal impedance-based inverse filtering of voiced sounds using neck surface acceleration. IEEE Trans. Audio Speech Lang. Process. 21, 1929–1939. 10.1109/TASL.2013.2263138 25400531 PMC4229092

[B61] Zañartu M. Mongeau L. Wodicka G. R. (2007). Influence of acoustic loading on an effective single mass model of the vocal folds. J. Acoust. Soc. Am. 121, 1119–1129. 10.1121/1.2409491 17348533

[B62] Zhang Z. (2020). Estimation of vocal fold physiology from voice acoustics using machine learning. J. Acoust. Soc. Am. 147, EL264–EL270. 10.1121/10.0000927 32237804 PMC7075716

